# Subdural haematomas drain into the extracranial lymphatic system through the meningeal lymphatic vessels

**DOI:** 10.1186/s40478-020-0888-y

**Published:** 2020-02-14

**Authors:** Xuanhui Liu, Chuang Gao, Jiangyuan Yuan, Tangtang Xiang, Zhitao Gong, Hongliang Luo, Weiwei Jiang, Yiming Song, Jinhao Huang, Wei Quan, Dong Wang, Ye Tian, Xintong Ge, Ping Lei, Jianning Zhang, Rongcai Jiang

**Affiliations:** 1 Department of Neurosurgery, Tianjin Medical University General Hospital, Tianjin, 300052 China; 2 Tianjin Neurological Institute, Key Laboratory of Post -Neuroinjury Neuro -repair and Regeneration in Central Nervous System, Ministry of Education and Tianjin, Tianjin, 300052 China; 3 Department of Geriatrics, Tianjin Medical University General Hospital, Tianjin, 300052 China

**Keywords:** Lymphatic vessel, Subdural haematoma, Drainage, Meningeal, Lymphangiogenesis

## Abstract

Subdural haematomas (SDHs) are characterized by rapidly or gradually accumulated haematomas between the arachnoid and dura mater. The mechanism of haematoma clearance has not been clearly elucidated until now. The meningeal lymphatic vessel (mLV) drainage pathway is a novel system that takes part in the clearance of waste products in the central nervous system (CNS). This study aimed to explore the roles of the mLV drainage pathway in SDH clearance and its impacting factors. We injected FITC-500D, A488-fibrinogen and autologous blood into the subdural space of mice/rats and found that these substances drained into deep cervical lymph nodes (dCLNs). FITC-500D was also observed in the lymphatic vessels (LYVE+) of the meninges and the dCLNs in mice. The SDH clearance rate in SDH rats that received deep cervical lymph vessel (dCLV) ligation surgery was significantly lower than that in the control group, as evaluated by haemoglobin quantification and MRI scanning. The drainage rate of mLVs was significantly slower after the SDH model was established, and the expression of lymphangiogenesis-related proteins, including LYVE1, FOXC2 and VEGF-C, in meninges was downregulated. In summary, our findings proved that SDH was absorbed through the mLV drainage pathway and that haematomas could inhibit the function of mLVs.

## Introduction

The lymphatic system plays an important role in modulating tissue homeostasis, excess fluid clearance and macromolecule and immune cell migration [3, 6, 38]. However, the central nervous system (CNS) was considered an organic system devoid of lymphatic vasculature until 2015, when two groups of researchers discovered lymphatic endothelial cells (LECs), which express classic lymphoid markers (LYVE 1, PROX 1, VEGF R3, FOXC 2 and podoplanin), and functional lymphatic vessels in the dura of rodents [1, 5, 24, 33]. It has also been demonstrated that meningeal lymphatic vessels (mLVs) drain brain interstitial fluid (ISF) and macromolecules, providing new insights into the clearance of waste in the CNS [5, 7, 23]. Subsequent studies have revealed that mLVs are responsible for the clearance of β-APP and tau, which are associated with neurological deficits in the context of Alzheimer’s disease (AD) [8]. This finding further demonstrates that mLVs play key roles in the clearance of intracranial waste.

Subdural haematomas (SDHs), the most common disease of the subdural space, progressively accumulate between the arachnoid and dura mater; SDHs consist of acute SDH, subacute SDH and chronic SDH (CSDH) [21, 32, 34]. CSDH is predicted to be one of the most common neurosurgical diseases, especially in individuals over 70 years old [21, 27, 28]. It has been demonstrated that CSDH can be absorbed spontaneously and that atorvastatin can promote this process [35, 40]. Despite the presence of multitudinous and dense inflammatory cytokines in CSDHs, only a few patients have demonstrated systemic inflammatory responses that include fever and increased white blood cell counts during the process of haematoma absorption [11, 19, 26], and most CSDH patients present with mild neurological symptoms [17]. These clinical phenomena evoke the question of how SDHs drain. If haematomas are absorbed through the vascular system, it remains unclear why no inflammatory responses or clinical symptoms are observed. In addition, the cerebrospinal fluid (CSF) of patients with CSDH is clear, and the cell counts in the CSF are also in the normal range, which indicates that CSDHs do not drain into subarachnoid CSF [20]. Based on the anatomical position of SDHs, adjacent to the mLVs [5, 21, 24], we hypothesize that haematomas in the subdural space may drain out of the cranial space through mLVs.

Previous studies have proven that deep cervical lymph nodes (dCLNs) represent an important site for the drainage of mLVs from intracranial structures to the extracranial lymphatic system [5, 23, 24]. Different substances, such as macromolecules, fluoresceins and Evans Blue (EB), can be detected in dCLNs when they are drained through mLVs [24, 43] . Furthermore, the drainage function of mLVs is significantly decreased after ligating deep cervical lymph vessels (dCLVs) [5, 43], suggesting that the presence of the pathway can be indirectly demonstrated by the detection of substances in dCLNs that are injected into the subdural space.

In this report, a modified subdural injection method and an SDH model were applied [35, 37]. Based on this, we demonstrated the drainage role of mLVs in SDHs by detecting whether substances (FITC-500D, autologous blood, A488- fibrinogen) injected into the subdural space could drain into dCLNs and by evaluating whether dCLV ligation surgery can slow the processes of SDH clearance. The effect of SDHs on the function of mLVs was also analysed by measuring the drainage rate and protein expression levels of lymphoendothelial-related molecules in meninges after establishment of the SDH model.

## Materials and methods

### Animals

Sprague-Dawley (SD) rats (9 to 10 weeks, 340 to 360 g, HFK Bioscience Co., Ltd., Beijing, China) and male ICR mice (12 weeks, 45 to 50 g, Vital River Laboratory Animal Technology Co., Ltd., Beijing, China) were housed in the animal facility of Tianjin Medical University General Hospital under a 12-h light/dark cycle in a temperature-controlled room (20 ± 2 °C) and provided food and water ad libitum. All experimental procedures involving the animals were reviewed and approved by the Tianjin Medical University Animal Ethics Committee (Tianjin, China). Experiments were reported according to the Animal Research: Reporting of in Vivo Experiments (ARRIVE) guidelines [10]. Common physiological states, such as the vasomotor, anaesthetic conditions, and positions, might be factors influencing intracranial material drainage. To avoid these factors in this study, we strictly kept the physiological/pathological statuses of experimental animals consistent during the experiment. Briefly, all the experimental animals were kept in the same suitable environment. For each individual experiment, we ensured that the test animals came from the same batch and had the same age and similar weight. During anaesthesia, all animals were given the same dose of chloral hydrate (300 mg/kg) to ensure that the animals’ anaesthetic states were similar. Animals that did not awake after anaesthesia and surgery were placed in the supine position on a thermostatic pad (38 °C) to maintain smooth breathing and constant body temperature. In the surgical segment of the experiment, all operations were standardized and consistent as much as possible, and the sham group animals were subjected to surgical injuries similar to those of the model groups to reduce bias. All animals were intraperitoneally injected with the same dose of ibuprofen to relieve pain after surgery and were kept in separate cages until they were sacrificed.

### Experimental procedures

**Fig. 1:**
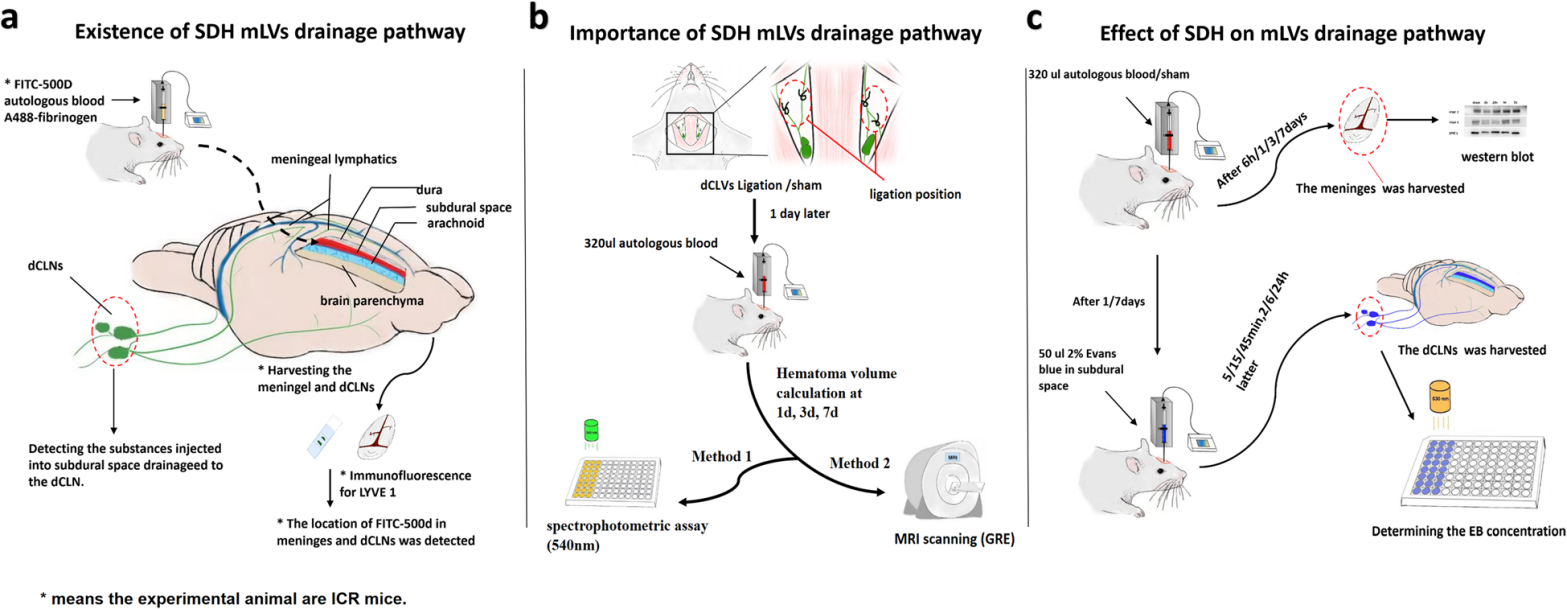
Schematic diagram of the experimental process. **a** Existance of SDH mLVs drainage pathway. **b** Importance of SDH mLVs drainage pathway. **c** Effect of SDH on mLVs drainage pathway

The study was divided into three parts. The aim of the first part was to prove the existence of the lymphatic drainage pathway from the subdural space into the extracranial lymphatic system and the role of mLVs. FITC-500D (Thermo Fisher, cat no. D7136), A488-fibrinogen (Thermo Fisher, cat no. F13191) and autologous blood were injected into the subdural space of the rats/mice, and drainage into the dCLNs was assessed (Fig. 1a). The second part was designed to prove that the drainage of SDH was dependent on the mLV drainage pathway. Thus, the dCLVs, which represented the key drainage route from the mLVs to the dCLNs, were ligated to observe the draining effect (Fig. 1b). The third part was designed to detect the effect of SDH on the function of mLVs. The EB stain (Sigma-Aldrich, cat no. E2129) was injected into the rats’ subdural space at different absorption stages of SDH. Then, the drainage curve of mLVs was drawn according to the concentration of EB in the dCLNs at different time points after the injection. The expression levels of lymphoendothelial-related proteins on the meninges were also detected at different stages of SDH (Fig. 1c).

### Subdural injection and SDH model

The SDH model and subdural injection method were modified from the SDH model used in our previous study [35, 37]. These methods allowed for the injection of autologous blood and other substances into the subdural space of rats/mice without damaging the brain parenchyma (Additional file 1: Figure S1 b and c). After anaesthetization with 70 mg/ml chloral hydrate (300 mg/kg), the rat’s hair was shaved. Then, the scalp was sterilized, and a 1.5-cm incision was made along the midline. Rats were then fixed on a stereotaxic frame, and a bone hole with a diameter of 1.2 mm was drilled 3 mm to the right and 0.5 mm forward from bregma to a depth of 1 mm, which created a hole. The dura was pricked with a sharp needle, and the puncture was enlarged with microscopic tweezers (DUMONT 5# F.S. T). Next, 320 μl of autologous blood was extracted from the femoral vein and slowly injected into the subdural space with a trace injection pump at a velocity of 60 μl/min with a 14 G conical indwelling needle, which was trimmed to just fit into the hole in the skull. The sham group was injected with the same volume of saline instead of autologous blood. The needle was held in place for 10 min to prevent leakage before it was removed. The bone hole was sealed with medical glue, and the scalp incision was closed. Ibuprofen (10 mg/kg) was injected intraperitoneally to relieve pain after the surgery.

The method of A488-fibrinogen, EB and FITC-500D injection was similar to that used to establish the SDH model. The specific experimental parameters are as follows: 20 μl of A488-fibrinogen was mixed with 300 μl of autologous blood (a total of 320 μl), and the subsequent injection process was exactly the same as that used for the SDH model. Next, 50 μl of 2% EB was subdurally injected in the same manner as autologous blood in the SDH model; 20 μl of 1% FITC-500D was injected into the subdural space of the ICR mice. This process required a 22G conical indwelling needle to cooperate with a 0.7 mm bone hole and a more delicate operation at the pricking dura stage.

Because the main objective of this study was to confirm the existence of an intracranial-extracranial lymphatic route from the subdural space to dCLNs, the rodent animal models applied in this experiment were maintained at a good status to avoid brain injury. The volumes injected into the subdural space of the rats and mice were limited to 320 μl and 20 μl, respectively, to prevent severe mass haematomas in the brain. However, the postoperative neurological functions of the animals were slightly changed, thus making the data related to neurological function difficult to reflect.

### TBI rat model

The traumatic brain injury (TBI) rat model was induced using a controlled cortical impact (CCI) device (eCCI-6.3 device, Custom Design & Fabrication) to provide a positive control for TUNEL staining (Additional file 1: Figure S1c). Briefly, the anaesthetized SD rats were subjected to a single cortical impact after creating a bone window on the parietal bone with a diameter of 5 mm. The impact parameters were as follows: velocity: 6 m/s, depth: 2 mm and dwell time: 250 ms. After 24 h of CCI beating, brain tissue was obtained, and sections frozen at a thickness of 10 μm were obtained for TUNEL staining.

### Ligation surgery of the dCLVs

Ligation surgery of the dCLVs was performed as previously described with a small modification [24]. This method can effectively slow down or block the main drainage pathway of mLVs through the dCLVs (Fig. 4a). In brief, after a 2-cm-long skin incision was made along the midline of the neck, the cervical fascia was cut layer by layer, and the thyroid gland was removed to the cephalic side. A small retractor was used to expose the anterior cervical muscle and sternocleidomastoid muscle. The dCLVs and dCLNs were further separated and ligated bilaterally with 5–0 sterile nylon thread (avoiding blood vessels and nerves). The rats in the sham group were subjected to the same procedure as those in the ligation group except for the ligation of the dCLVs. The rats were raised separately after the ligation surgery and underwent SDH surgery the next day.

### H&E staining

H & E staining of brain and dCLN tissues of SDH rats was performed using coronal paraffin sections with a thickness of 7 μm, and the dyeing procedure was conducted according to the instructions of the H&E staining kit (Solarbio, cat no. G1120). In particular, coronal sections of brains require retention of the dura for more convenient visualization of the SDH.

### Immunofluorescence (IF)

Because of the unavailable high-quality antibodies to rats, ICR mice were used for immunofluorescence staining to label the lymphatic vessels on the meninges and dCLNs. Whole-mount meninges and dCLNs were obtained after FITC-500D was injected into the subdural space for 6 h. After fixation with 4% PFA, the meninges were washed with PBS to remove the FITC-500D attached to the surfaces, and the dCLNs were cut into 10-μm-thick sections with frozen slicers (Licia). Then, the meninges and dCLN sections were incubated with 2% BSA, 2% goat serum, 0.2% Triton X-100, and 0.05% Tween 20 in PBS at room temperature for 1.5 h. Subsequently, they were incubated with primary antibodies (rabbit anti-LYVE1 pAB (1:100, Abcam, cat no. ab14917)) at 4 °C overnight. After being sufficiently washed with PBS, the tissues were incubated with fluorescent secondary antibodies (Alexa Fluor 594 donkey anti-rabbit IgG (1:1000, Invitrogen, cat no. A21207)) for 1 h at RT. Then, the meninges and dCLN sections were incubated with DAPI for 15 min (1:20000, Invitrogen, cat no. D1306), mounted with glass coverslips and imaged with an inverted fluorescence microscope (Olympus).

Ten-micron-thick brain coronal sections were collected and double stained with TUNEL and an anti-NeuN mAB to determine whether the SDH model caused cortex damage. The staining protocol was the same as that described above. A rabbit anti-NeuN mAB (EPR12763) (1:200, Abcam, cat no. ab177487) and an Alexa Fluor 594 donkey anti-rabbit lgG (1:1000, Invitrogen, cat no. A21207) were used as the primary antibody and fluorescent secondary antibody, respectively. After incubating the slides with the fluorescent secondary antibody, 30 μl of TUNEL reaction mixture solution (Roche, cat no. 11684817910) was added to each section, and the slides were incubated for 1.5 h at 37 °C. After washing with PBS, the sections were incubated with DAPI (Abcam; cat no. ab104139) and mounted with cover glass.

### Quantification of haematoma volumes

The residual volumes of SDH at the 6th hour, 3rd day and 7th day after surgery were calculated by two methods (haemoglobin quantification and MRI scan) in this study. Haemoglobin quantification was carried out with a spectrophotometric assay according to the methods in previous studies [25]. The meninges with haematomas were collected as completely as possible after euthanasia and perfused with PBS. Then, the tissue was ground, ultrasonicated and centrifuged at 13000 RPM for 30 min with 1.5 ml of PBS. Then, 0.4 ml of supernatant from each sample was mixed with 1.6 ml of Drabkin’s reagent (Sigma-Aldrich, cat no. D5941) for 15 min at RT, and the mixture was added to a 96-well plate at a volume of 200 μl per well. The absorbance value of each well was detected at a wavelength of 540 nm using an automatic microplate analyser. Blood (320 μl) was obtained from the inner canthus veins of three normal SD rats, and 8 serial dilutions were made with PBS (haematoma volumes of 320 μl, 160 μl, 80 μl, 40 μl, 20 μl, 10 μl, 5 μl and 2.5 μl) to establish a standard curve. After the establishment of the standard curve, the residual haematoma volumes in each group were calculated with Excel.

The changes in haematoma volumes of dCLV ligation/control SDH rats were also measured with a 3 T MRI scanner (MR750, GE Healthcare, Chicago, IL, USA) as previously described [37]. SDH rats in the dCLV ligation/control groups underwent MRI scanning at 6 h, 3 d and 7 d after SDH surgery. The rats were fixed on a four-channel phased-array small animal coil (phi =6 cm, Magtron, Shenzhen, China) in the supine position for MRI after anaesthesia. Each rat’s head was scanned from the olfactory bulb to the cerebellar hemisphere with a gradient-recalled echo (GRE) sequence. The parameters for GRE scanning were as follows: a repetition time (TR) of 200 ms, an effective echo time (TE) of 6 ms, and a field of view (FOV) of 18 × 18 mm with a matrix of 512 × 512. Twenty-five slices were analysed, each with a slice thickness of 1.0 mm. Due to the limited resolution of MRI scans and the special shape of SDHs (thin crescent), it is inaccurate to calculate the true haematoma volume via scanning images with tools or software. Therefore, the percentage of residual haematoma volume in the data graph was used to evaluate the absorption of haematoma in each group of SDH rats. Briefly, based on the consistent pixel density of all MRI images in this study, we used photoshop CS6 (Adobe) to circle the haematoma areas of the scanned images and obtain the number of pixels. Then, the numbers of pixels in the haematoma regions at each scan level were added as the virtual equivalent haematoma volume. Then, the percentage of residual haematoma volume = haematoma volume at a specific time point after SDH modelling/SDH haematoma volume measured immediately after SDH modelling.

### Evans blue (EB) injection and quantification

On the 1st and 7th day after the SDH model was established, 50 μl of 2% EB was injected into the subdural space of SDH/sham rats to detect the regularity of mLV drainage in different stages of haematoma formation. Both sides of the dCLNs were harvested after the rats were euthanized and perfused with PBS at 5 min, 15 min, 45 min, 2 h, 6 h and 24 h after EB injection. Then, the EB in the dCLNs was extracted by incubating the tissue with formamide (5 μl per milligram of tissue) for 24 h in a 60 °C water bath. After centrifugation at 3000 RPM, 100 μl of supernatant was collected and added to a 96-well plate for quantification of the EB concentration. The optical density (OD) values were measured at a wavelength of 620 nm with a microplate reader (Thermo Fisher). Finally, the standard curve and EB concentrations were analysed with Excel.

### Western blot analysis

Western blot was used to analyse the lymphoid-associated protein changes in meninges at different time points after SDH/sham surgery. Briefly, after euthanasia and perfusion with cold PBS, the rat meninges were harvested, and total protein was extracted with RIPA lysis buffer and 1% PMSF. The protein was separated by 10% SDS-PAGE at 120 V for 90 min, and the separated proteins were transferred to PVDF membranes. Then, the PVDF membranes were blocked and incubated with primary antibodies (rabbit anti-LYVE1 (1:1000, Abcam, cat no. ab183501); rabbit anti-PROX1 (1:1000, Abcam, ab199359, Cambridge, UK); rabbit anti-VEGF receptor 3 (1:200, Abcam, cat no. ab27278); mouse anti-VEGF C mAB, (1:500, Abcam, cat no. ab106512); mouse anti-FOXC2 (1:500, Sigma-Aldrich, cat no. WH0002303M2); and mouse anti-β actin mAb (1:1000, ZSGB-BIO, cat no. TA-09)) on a shaker overnight at 4 °C. After being sufficiently washed, the membranes were incubated with horseradish peroxidase-conjugated secondary antibodies (1:5000, ZSGB-BIO, cat no. ZB-2301 and cat no. ZB-2305) for 1 h at room temperature. A ChemiDoc imaging system (Bio-Rad) was used to visualize the membranes, and ImageJ software was used to calculate the grey values of the bands.

### Statistics

All the data were analysed with unpaired t tests using SPSS software (version 22.0, IBM). All the values are shown as the mean ± SD. A *p* value less than 0.05 was considered to indicate statistical significance.

## Results

### Macromolecular fluorescein in the subdural space drained into dCLNs through the mLVs

**Fig. 2:**
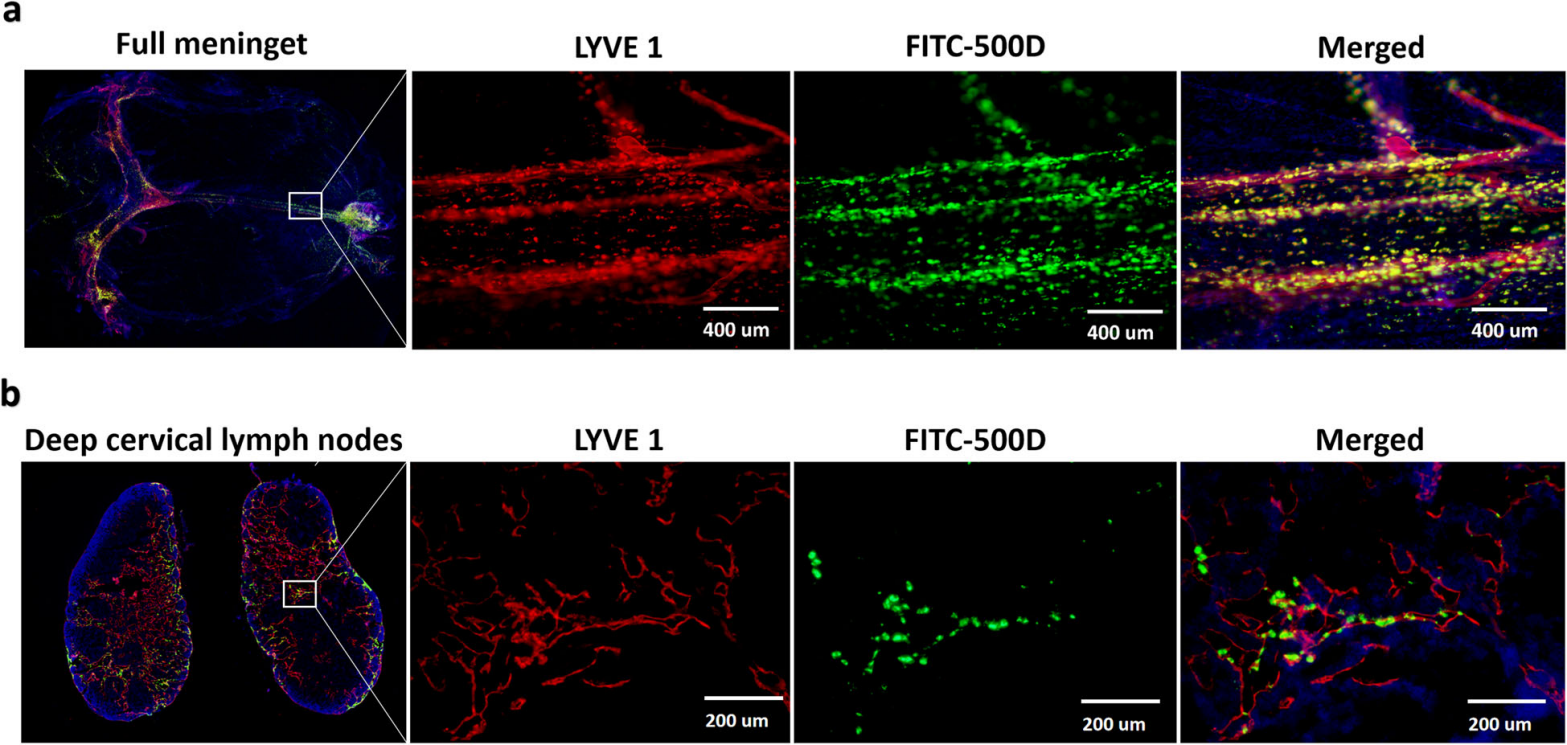
The subdural fluorescent macromolecule drainage pathway colocalized with lymphatics in the meninges and dCLNs. **a** Immunofluorescence images of mouse mLVs (LYVE 1+). The merged channel shows that FITC-500 D colocalizes with LYVE 1+ mLVs near the superior sagittal sinus. **b** Immunofluorescence images of the mouse lymph sinus of deep cervical lymph nodes (LYVE 1+). Fluorescence colours of (**a**) and (**b**): LYVE 1, red; FITC-500 D, green; DAPI, blue. *n* = 3 (mice). The merged channel shows that FITC-500 D appears in the lymph sinus

To test how the macromolecular waste in the subdural space was cleared, A488-fibrinogen, EB and FITC-500D were injected into the subdural space. Since the tracers injected into the parenchyma and CSF were drained into the dCLNs, we used a microscope to inject the tracers precisely into the subdural space without damaging the parenchyma. TUNEL staining, a well-known experiment assessing parenchymal damage, demonstrated that our SDH model had no effect on the brain parenchyma and that the tracers injected into the subdural space did not enter the CSF or parenchyma (Additional file 1: Figure S1). Immunofluorescence staining and fluorescence imaging of the meninges and dCLNs of mice showed the lymphatic drainage channel of FITC-500D in the subdural space. On the meninges, the FITC signal was observed inside vessel-like structures along with the sinus but not inside the sinus and was mainly colocalized with LYVE 1 (+), a marker of LECs (Fig. 2a). Similarly, the FITC signal was found in only the lymph sinus (LYVE 1+) of dCLNs (Fig. 2b). These results proved that the macromolecules in the subdural space indeed drained into the dCLNs through the mLVs.

### Red blood cells and fibrinogen were drained into the dCLNs

**Fig. 3:**
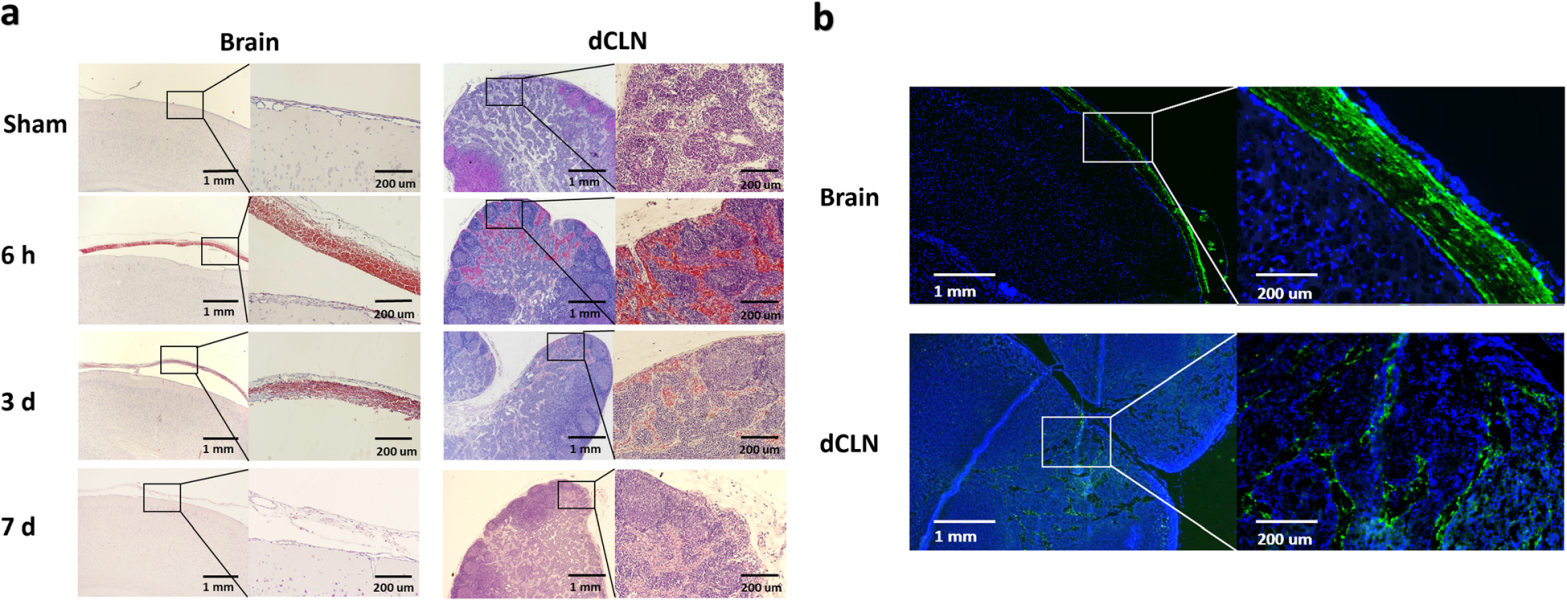
SDH drained into dCLNs. **a** H & E staining images of brain coronary and dCLN sections in the sham and SDH groups at 6 h, 3 d and 7 d after SDH establishment. n = 3/group. **b** Fluorescence images of rat brain coronary and dCLN sections at 6 h after the injection of A488-fibrinogen mixed with autologous blood into the subdural space. Green fluorescence signals were detected in the rat subdural space and dCLNs. Fluorescence colours: A488-fibrinogen, green; DAPI, blue. *n* = 3

To further confirm whether red blood cells and fibrinogen could be drained into dCLNs, H & E staining was performed, and A488-fibrinogen was injected into the subdural space. H & E staining showed that many red blood cells accumulated in the dCLNs of the SDH rats only 6 h after the operation, and the quantity of red blood cells decreased with haematoma absorption (*n* = 3/group) (Fig. 3a). Nearly no red blood cells were observed in the dCLNs of the sham rats (Fig. 3a). Additionally, A 488-fibrinogen also drained from the subdural space to the dCLNs (*n* = 3) (Fig. 3b). These results confirmed that the components of SDH, including red blood cells and fibrinogen, could also be drained by the lymphatic pathway.

### Ligation of cervical lymphatic vessels inhibited SDH absorption

**Fig. 4:**
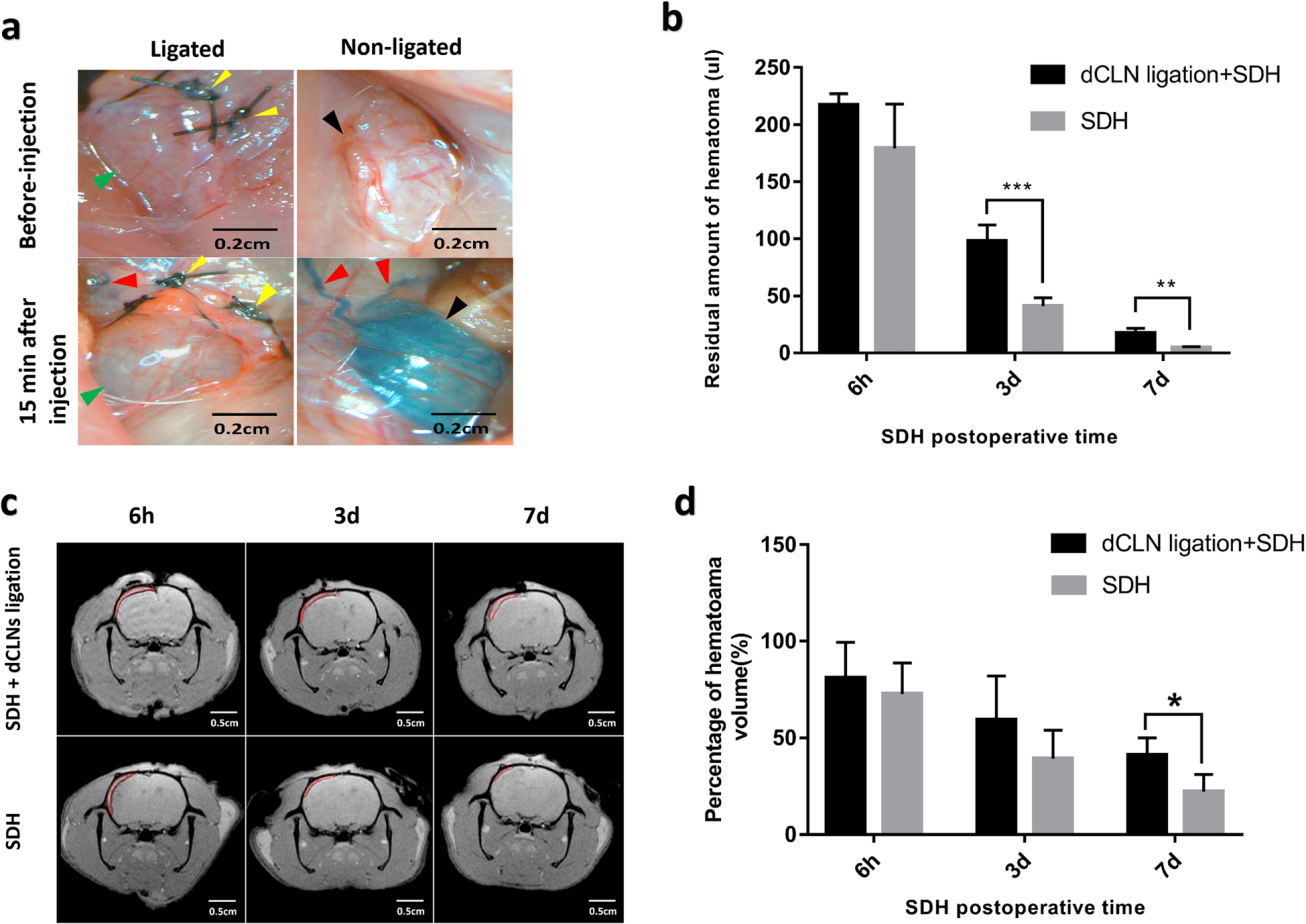
Ligation of dCLVs inhibited the clearance of SDH. **a** Image displaying the effectiveness of dCLV ligation surgery (to observe the bluish colour of dCLNs of ligated and nonligated sides 15 min after the injection of EB into the subdural space). The green and black arrows indicate dCLNs on the ligated side and the nonligated side, respectively. The yellow and red arrows indicate the ligation points and dCLVs, respectively. Ligation of dCLVs obviously blocked the downward drainage of EB, which was injected into the subdural space. **b** The residual haematoma volumes of SDH rats at 6 h, 3 d and 7 d after SDH surgery in the dCLV ligation group and control group as measured by haemoglobin quantification. *n* = 5/group for each time point. **c** Representative MRI images of SDH rats. The haematoma areas are outlined in red. **d** Percentage of residual haematoma volumes according to MRI data of SDH rats in the ligation and control groups (percentage of residual haematoma volume = haematoma volume at a specific time point after SDH modelling/SDH haematoma volume measured immediately after SDH modelling). *n* = 5/group. The data in (**b**) and (**d**) are presented as the mean ± SD. *P < 0.05, ** *P* < 0.01, *** *P* < 0.001

To test the hypothesis that SDH was drained into the dCLNs, dCLVs were ligated, and the haematoma volume was quantified by haemoglobin quantification and MRI scanning. The residual haematoma volumes measured by spectrophotometric assay in the dCLV ligation group were significantly greater than those in the control group on the 3rd and 7th days after SDH surgery (*n* = 5/group; *P* < 0.05) (Fig. 4b). MRI was also performed to assess the haematoma volume differences between the SDH rats with and without ligated dCLVs (Fig. 4c). The results showed that the haematoma volumes were significantly greater in the SDH rats with ligation than in those subjected to sham surgery on the 7th day (*n* = 5/group; *P* < 0.05) (Fig. 4d), which further illustrated the importance of the lymphatic drainage pathway for SDH absorption.

### SDH inhibited the drainage efficiency of mLVs and modulated lymphangiogenesis

**Fig. 5:**
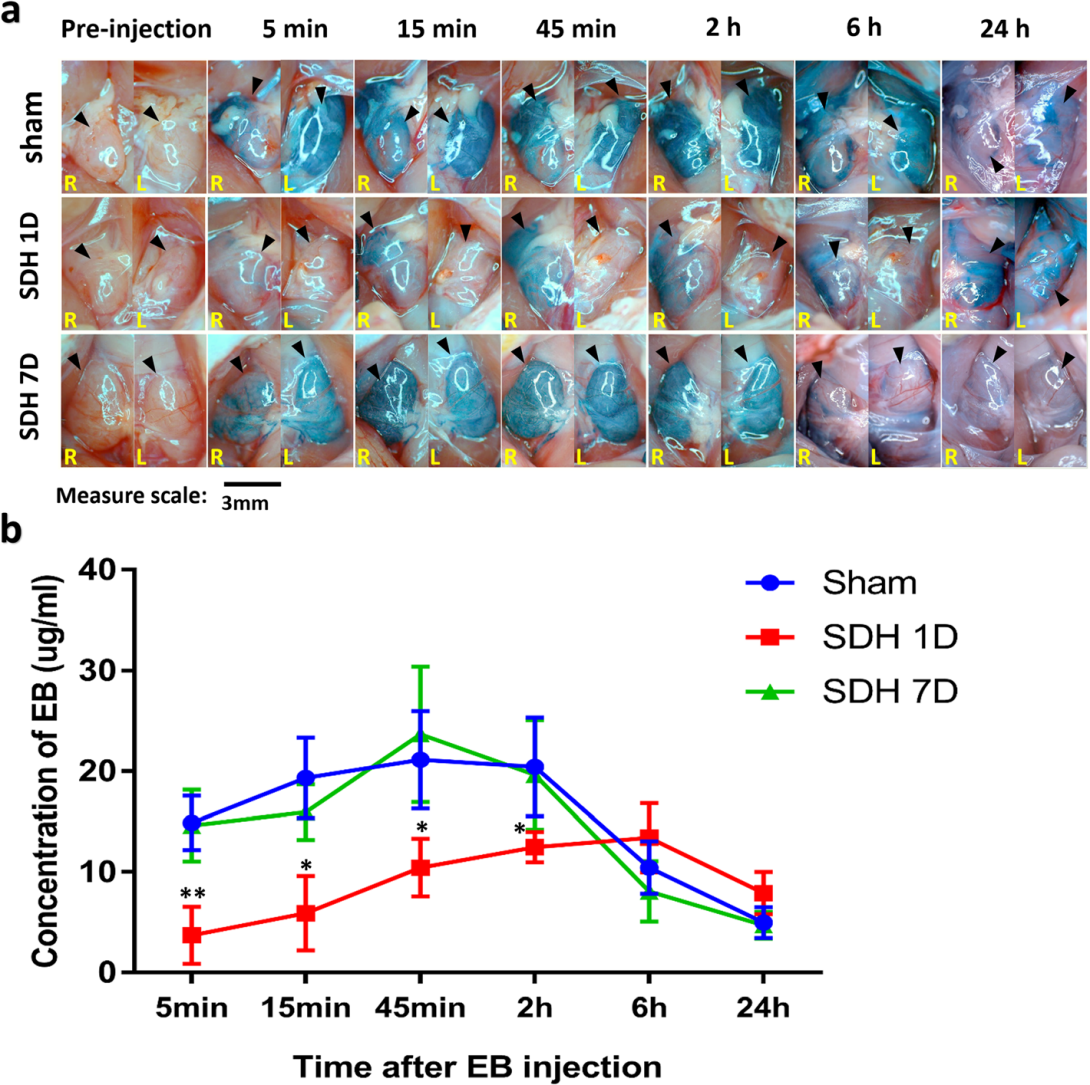
The haematoma inhibited EB drainage into the dCLNs. **a** Representative images of dCLNs in the Sham group, SDH 1D group and SDH 7D group at different time points after EB injection (SDH 1D and SDH 7D represent EB injected on the 1st and 7th day after SDH modelling). The black arrows indicate the dCLNs. **b** The EB concentration in the dCLNs at different timepoints after EB injection was quantified by absorption spectrophotometry. The data are presented as the mean ± SD. The EB concentration in the dCLNs of SDH 1D rats was significantly lower than that in the sham group rats at 5 min, 15 min, 45 min and 2 h after the EB subdural injection. However, there were no significant differences in the EB concentration in dCLNs of SDH 7D and sham group rats at any time point after injection. *p < 0.05, **p < 0.01, compared with the sham group, *n* = 4/group for 7 time points

**Fig. 6:**
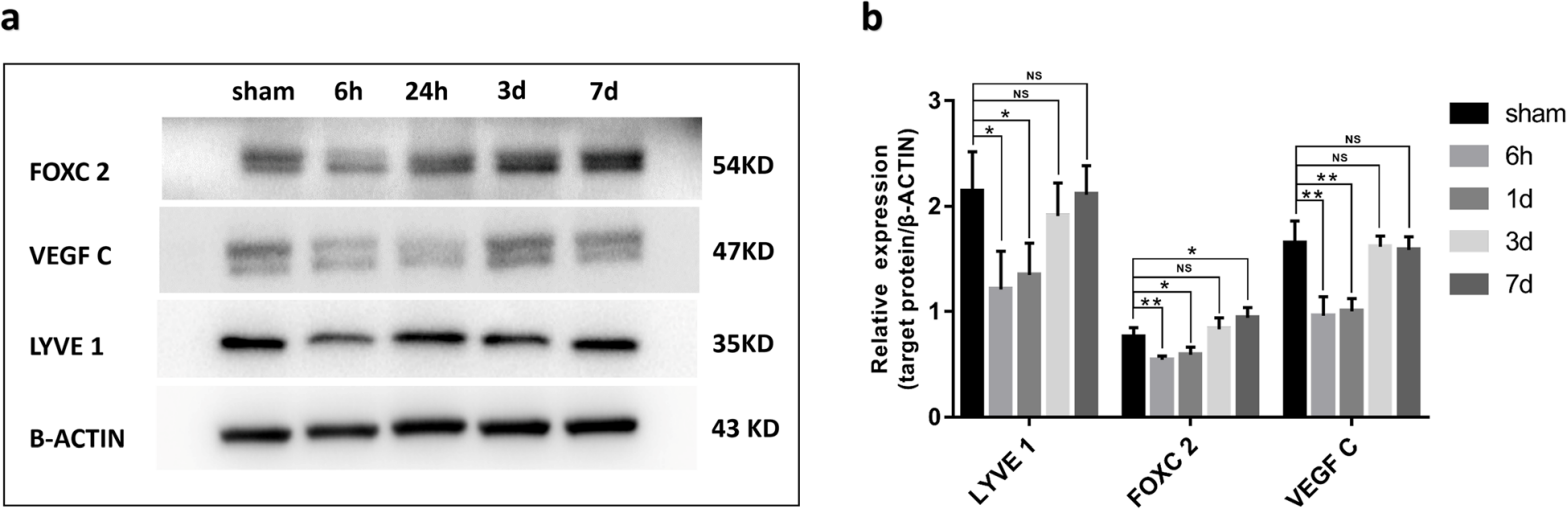
Effect of SDH on the expression of lymphoid markers in the meninges. **a** Representative Western blot images of LYVE 1, FOXC 2 and VEGF-C in the meninges of rats in the sham and SDH groups at 6 h, 24 h, 3 d and 7 d after SDH establishment. B-actin was used as an internal reference protein. **b** Quantification of LYVE 1, FOXC 2 and VEGF-C expression. The expression levels of these three lymphatic protein markers decreased in the SDH group at 6 h and 1 d after the modelling and returned to the baseline on days 3 and 7. The data are presented as the mean ± SD. *P < 0.05, ** *P* < 0.01, ^**NS**^*P* > 0.05, *n* = 4/group

Whether haematomas could modulate the drainage efficiency of mLVs was still unknown. To assess the drainage efficiency, EB was injected into the subdural space on the 1st (SDH 1D) and 7th days (SDH 7D) after SDH modelling. The concentration of EB, which was drained from subdural spaces into dCLNs, was measured in the dCLNs of sham rats and SDH 1D and SDH 7D rats at 5 min, 15 min, 45 min and 2 h after EB injection. In the SDH 1D group, the EB concentration was significantly lower than that in the sham group, which indicated that the lymphatic drainage efficacy was inhibited (Fig. 5a and b). However, in the SDH 7D group, the efficacy of the drainage rate from the subdural space to the dCLNs in the SDH rats was similar to that in sham rats (Fig. 5a and b). Western blot analysis of meninges showed that the expression of lymphatic protein markers (LYVE 1, VEGF C and FOXC 2) was decreased in the SDH group at 6 h and 1 d after SDH formation (*n* = 4/group) (Fig. 6a and b). Upon haematoma absorption, the expression levels of these proteins returned to the baseline (*n* = 4/group) (Fig. 6a and b). These results suggested that the haematoma could decrease the mLV drainage ability and inhibit the production and function of meningeal lymphatic endotheliocytes. This effect was recovered with the gradual absorption of the haematoma.

## Discussion

To the best of our knowledge, this study is the first to demonstrate the existence of a lymphatic drainage pathway connecting the subdural space, mLVs and dCLNs. The blood component of fibrinogen, the macromolecule FITC-500, could be drained from the subdural space to the dCLNs. This intracranial-extracranial pathway is essential for the removal of subdural macromolecular substances into the peripheral circulation. Although the existence of a circulatory pathway connecting the subdural cavity to the peripheral circulation was not completely ruled out in this work, the decreased drainage efficacy after cervical lymphatic vessel ligation strongly indicates that a direct pathway from the mLVs to the extracranial lymphatic system plays a key role in intracranial waste clearance. Because the lymphatic system is independent of the circulation, this new finding of an intracranial-extracranial lymphatic drainage pathway also provides a reasonable explanation for the lack of inflammatory reactions during clinical CSDH formation and atorvastatin-driven haematoma elimination [36, 40].

The decreased drainage efficacy of this lymphatic pathway in SDH rats compared with sham rats might have resulted from the direct injury to the mLVs caused by the modelling, which was consistent with the expression changes in lymphangiogenesis markers in the meninge. However, an indirect impact of increased intracranial pressure (ICP) on lymphatic drainage function should also be considered. Although there is no evidence that ICP plays a role in intracranial-extracranial lymphatic drainage, the impact of ICP on the resolution of cerebral oedema and hydrocephalus is well known [22, 31]. It is essential to further explore the role of ICP in the elimination of SDH.

The expression changes in lymphangiogenesis markers in the dura (LYVE1, FOXC2 and VEGF C) after SDH formation and during SDH elimination were remarkable. LYVE1 is a major marker of the lymphatic endothelium, and its expression is positively correlated with LEC quantity. VEGF C promotes the generation of LECs, which then migrate to form primary lymphatic vessels. FOXC2 is the key factor promoting the maturation of primary lymphatic vessels, which enables their performance of normal functions [13, 14, 29]. The existence of haematoma resulted in a decreased drainage efficacy of mLVs and decreased expression levels of LYVE 1, VEGF C and FoxC2. When the haematoma gradually shrank, the expression levels of these proteins returned to the baseline, indicating that the formation of SDH inhibits dural lymphangiogenesis and thus reduces the haematoma drainage. The basic function of the lymphatic system is to remove waste substances [4, 6]. Kipnis et al. demonstrated that mLVs are responsible for the clearance of β-APP and Tau, which are macromolecular waste products that are associated with neurological deficits in AD [8]. Therefore, the regulation of dural lymphangiogenesis might be a new therapeutic strategy for intracranial disease caused by intracranial macromolecular waste accumulation.

The glymphatic system is also called convective influx system or perivascular space, which is the space between the capillary endothelium and the layer of pericytes and astrocyte endfeets together with their basal laminae [12, 16, 18]. It responsible for the clearance of macromolecular and wastes in ISF and CSF out of the CNS [12, 16, 18, 30]. Intracranial bleeds, including TBI, intracerebral haemorrhage (ICH) and subarachnoid haemorrhage (SAH), release broken blood cell debris, toxic free radicals, inflammatory cytokines, microvesicles and waste macromolecules [9, 39, 41, 42], which is accompanied by impaired glymphatic system structures and functions [16]. Glymphatic system injury results in a decreased drainage rate of harmful intracranial substances after these diseases and thus exacerbates secondary brain damage. Past research has shown that tracers injected into the CSF enter and leave the brain along separate periarterial basement membrane pathways, It means that CSF may be a suitable route for delivery of drugs for neurological diseases therapies [2].

It has been confirmed that both the glymphatic system and mLVs drain macromolecular waste into dCLNs [5, 15]. Although there are no data showing a direct connection between the glymphatic system and mLVs, the fact that these two waster clearance pathways share the same destination indicates that they are connected. In this study, we found that the macromolecular wastes in the subdural space were also drained into the dCLNs through the mLVs, suggesting that the glymphatic system and mLVs may be connected through the subdural space. These findings imply that the blood cell debris and macromolecular wastes associated with brain injury (e.g., TBI, ICH, and CSDH) might be cleared through the glymphatic system entering into the adjacent meninges lymphatic system and finally into the extracranial lymphatic system. This hypothesis led us to think that, compared with those associated with deep brain injuries, harmful substances in injuries located near the dural or cistern may be much easier to drain out of the CNS since the mLV travel distance is shorter.

The discovery of the intracranial-extracranial lymphatic drainage pathway provides a good explanation for the lack of systemic reactions during CSDH absorption in conservative treatment. The strategy to enhance the drainage function of the intracranial-extracranial lymphatic system through either physical or pharmaceutical methods will provide new avenues for brain injury treatment.

This study found that the formation of SDH can impair the drainage efficiency and expression levels of related proteins in mLVs. However, which components of SDHs and the mechanisms by which SDHs affect the function of mLVs still need to be elucidated, which is the main direction of our next research study.

## Conclusions

The present study demonstrated that substances in the subdural space could also be drained into dCLNs through mLVs and confirmed the important role of this pathway in the process of SDH clearance. In addition, the formation of SDH can impair the drainage efficacy and expression of lymphangiogenesis-related proteins in mLVs.

## Supplementary information

**Additional file 1: Figure S1.** Process and schematic diagram of the rat SDH model. The modified SDH model was established without injury to the brain parenchyma. (a) Schematic diagram of the SDH model. (b) H & E staining of brain tissue sections from SDH rats showing large numbers of blood cells (blue arrows) located between the dura (yellow arrow) and arachnoid (white arrow); the black arrows show the pia and parenchyma. (c) Immunofluorescence images of brain tissue sections from rats in the sham group, SDH group and TBI group (positive control) 24 h after model establishment. Fluorescence colours: NeuN, red; TUNEL, green; DAPI, blue. *n* = 3/group. Results and description: SDH is located entirely between the dura and arachnoid membrane without leakage to the CSF or impairment of the adjacent parenchyma. To ensure that the model haematoma was located in the subdural space without leakage to the CSF or injury to the brain parenchyma, a microscope was utilized to precisely direct the establishment of the SDH model. The haematoma in this rat model was thus situated between the dura and arachnoid (Figs. s1 a and b). Neuronal apoptosis always indicates brain tissue injury. Immunofluorescence and TUNEL staining were applied and showed that the number of apoptotic neurons was comparable between the SDH rats and sham rats, and apoptotic cells were rarely observed (Fig. s1 c). This result suggests that SDH modelling causes virtually no damage to the brain parenchyma. Large numbers of apoptotic cells were observed in brain tissue sections of rats subjected to traumatic brain injury (TBI), which were used as positive controls (Fig. S1 c).

## Data Availability

The data sets generated during the current study are available from the corresponding authors on reasonable requests.

## Abbreviations

AD: Alzheimer’s disease
CCI: Controlled cortical impact
CNS: Central nervous system
CSDH: Chronic subdural hematoma
CSF: Cerebrospinal fluid
dCLN: Deep cervical lymph node
dCLV: Deep cervical lymphatic vessel
EB: Evans Blue
ISF: Interstitial fluid
LEC: lymphatic endothelial cell
mLV: Meningeal lymphatic vessel
MRI: Magnetic resonance imaging
SDH: Subdural hematoma
TBI: Traumatic brain injury
TUNEL: TdT-mediated dUTP Nick-End Labeling
