# Supplementary figures and images for: Subdural haematomas drain into the extracranial lymphatic system through the meningeal lymphatic vessels
Source: Acta Neuropathol Commun. 2020 Feb 14;8:16. doi: 10.1186/s40478-020-0888-y (PMC7023797; doi:10.1186/s40478-020-0888-y)

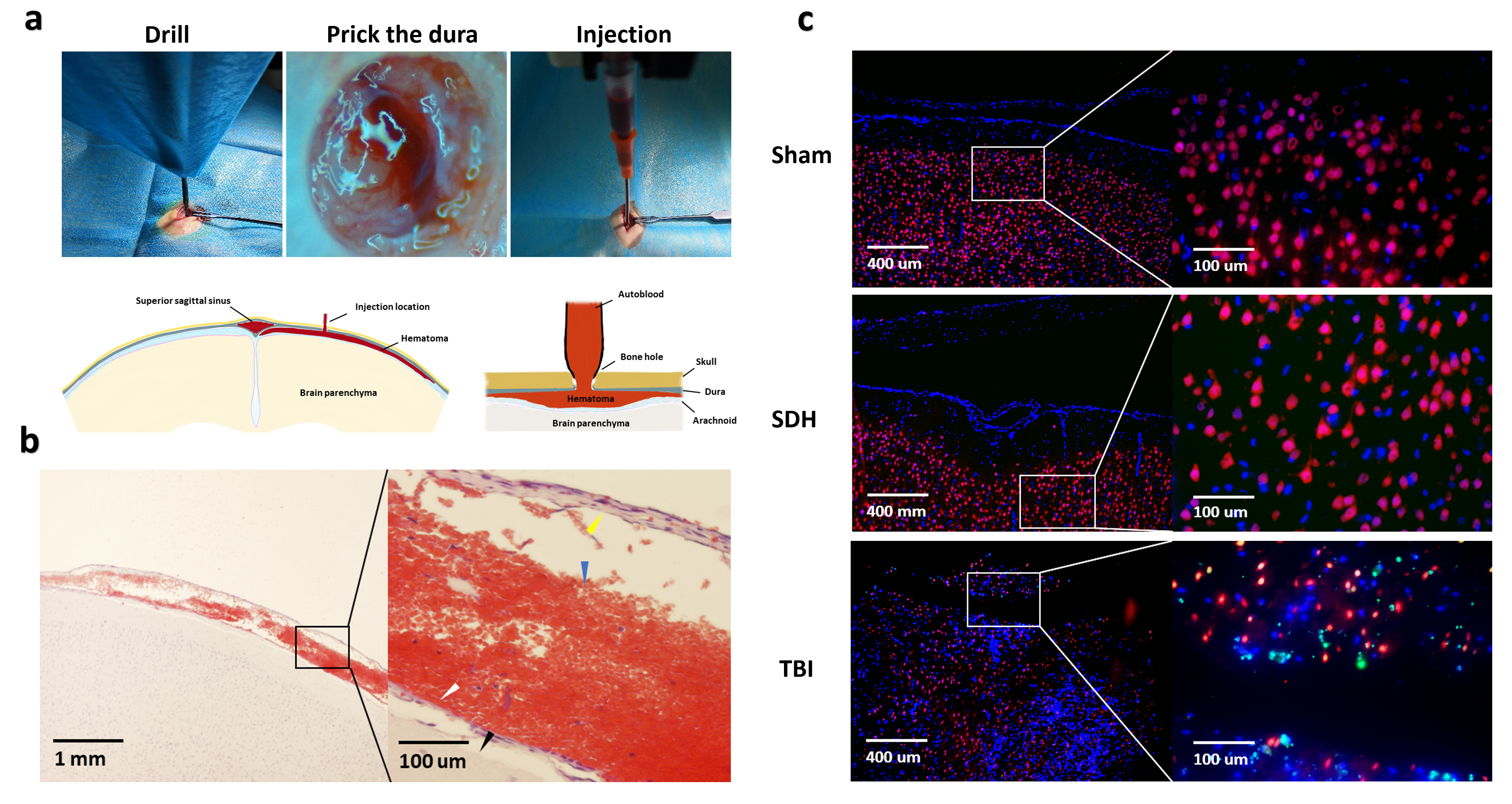

Supplement: Supplementary file 1 — Additional file 1: Figure S1. Process and schematic diagram of the rat SDH model. The modified SDH model was established without injury to the brain parenchyma. (a) Schematic diagram of the SDH model. (b) H & E staining of brain tissue sections from SDH rats showing large numbers of blood cells (blue arrows) located between the dura (yellow arrow) and arachnoid (white arrow); the black arrows show the pia and parenchyma. (c) Immunofluorescence images of brain tissue sections from rats in the sham group, SDH group and TBI group (positive control) 24 h after model establishment. Fluorescence colours: NeuN, red; TUNEL, green; DAPI, blue. n = 3/group. Results and description: SDH is located entirely between the dura and arachnoid membrane without leakage to the CSF or impairment of the adjacent parenchyma. To ensure that the model haematoma was located in the subdural space without leakage to the CSF or injury to the brain parenchyma, a microscope was utilized to precisely direct the establishment of the SDH model. The haematoma in this rat model was thus situated between the dura and arachnoid (Figs. s1 a and b). Neuronal apoptosis always indicates brain tissue injury. Immunofluorescence and TUNEL staining were applied and showed that the number of apoptotic neurons was comparable between the SDH rats and sham rats, and apoptotic cells were rarely observed (Fig. s1 c). This result suggests that SDH modelling causes virtually no damage to the brain parenchyma. Large numbers of apoptotic cells were observed in brain tissue sections of rats subjected to traumatic brain injury (TBI), which were used as positive controls (Fig. S1 c). [file 40478_2020_888_MOESM1_ESM.tif]
